# The Art of Keeping Clean

**Published:** 1921-03-19

**Authors:** 


### The Art of Keeping Clean.

Every schoolboy knows—at least, every schoolboy who has been spanked for not washing behind his ears knows—that cleanliness is not merely a duty, but an art. It is strange that, being "next to godliness," it should be a duty which seems so unnatural, but the fact is that one of the most difficult acquirements of the young is to learn how to keep himself clean. Class distinctions do not count for much in this matter. Anyone who thinks that children have a natural desire to be clean can be confuted by the horrible filthiness of some of the children in the nearest slum, but let him inquire at a first-class public school, and he will learn that even among the children of our most favoured classes the price of cleanliness, like that of liberty, is eternal vigilance. Sir Almroth Wright, in his famous attack on washing, at least pointed out the other side of the question, but the real moral of his attack is one that many of our army of doctors realised as they had never done before—that cleanliness, like beauty, is often only skin deep, and frequently fails to go so far.

Certainly this could be said of many of the men who came up for medical examination during the war. Among other causes of this deplorable state of things two stand out. Bad training is generally recognised. Another factor is that cleanliness costs money. Not much, perhaps, but in the straitened budget of many a working-class home the expenditure on soap (often lavishly wasted by children), towels (treated with incredible carelessness), tooth-brushes (which need to be constantly renewed if they are not to be worse than useless), and baths (which call for coal, only this week subjected to a rise in price) is a matter of serious concern to the housewife and mother. But all our insistence on the almost moral virtue of soap and baths has not brought home to our general population the thorough-going nature of real cleanliness. One of the most painful things to an Indian or a Japanese who comes among us is the offensive odour of an English crowd—not a picture-palace crowd or a mob confined in fetid conditions, but the ordinary cold-bathing, exercising Englishman. The politeness of Orientals forbids them remarking on it, and when challenged they are loath to own up to a feeling which they regard as reflecting most seriously upon us. But no man can rise higher than his nose will let him, and the fact remains that the most cleanly of Englishmen is, according to their standard and their sense of smell, palpably unclean. They are burnt in the sun and laved in constant bathings of a frequency and a stringency largely dictated by religious conceptions of duty and far beyond our own. It would be ludicrous for a modern Englishman to seek physical salvation along their lines. But the moral is clear.

Cleanliness is a deeper thing than we have often thought. Our insistence upon its outward forms has led many to think that, having conformed in those respects, they have done all. But it is far more necessary to have a clean mouth than a clean face. That can be secured not only by the use of tooth-brushes and mouth-washes, but by eating suitable food and by eating food in due order, following clinging and sticky food by astringent and cleansing articles of diet. The same argument applies to the stomach. It is not merely the selection of food that makes for cleanliness. It is the use of food in its proper order and due sequence, and also the provision of periods of time for the body to achieve its natural ablutions.

Let us revert then to the cleansing of the skin. An epidermis only surface-cleaned is not cleansed at all. Dirt lies in the skin unless removed, and it is pitiful that many people never regularly perspire, but attempt to make up for their deficiency in that respect by our over-vaunted hot weekly bath. So inverted are our ideas that to perspire is often regarded as something much worse than having a dirty face, whereas, rightly performed and rightly considered, sweating is to washing as work is to a hobby. It should be the duty of all those concerned in any way with the health of the public to lose no chance of instilling this deeper meaning of cleanliness and pointing out by example and precept how cheaply by means of exercise and cold water the best results can be attained.

